# The efficacy and safety of velagliflozin over 16 weeks as a treatment for insulin dysregulation in ponies

**DOI:** 10.1186/s12917-019-1811-2

**Published:** 2019-02-26

**Authors:** A. Meier, M. de Laat, D. Reiche, D. Fitzgerald, M. Sillence

**Affiliations:** 1 Earth, Environmental and Biological Sciences School, Queensland University of Technology (QUT), Brisbane, Queensland 4000 Australia; 2 Boehringer Ingelheim Vetmedica, 55218 Ingelheim am Rhein, Germany

**Keywords:** Insulin, Laminitis, Equine metabolic syndrome, Sodium-glucose linked transport inhibitor

## Abstract

**Background:**

A previous six-week (wk) study demonstrated the potential of the sodium-glucose linked transport inhibitor velagliflozin as a novel treatment for equine insulin dysregulation. The present study examined the safety and efficacy of velagliflozin over 16 wk. of treatment, and over 4 wk. of withdrawal. Twenty-four insulin dysregulated ponies were selected, based on their hyper-responsiveness to a diet challenge meal containing 3.8 g non-structural carbohydrates (NSC)/kg bodyweight (BW). Ponies with serum insulin > 90 μIU/mL either 2 or 4 h after feeding were enrolled, and randomly allocated to receive either velagliflozin (0.3 mg/kg BW orally once daily, *n* = 12), or a placebo (*n* = 10–12) for 16 wk. The subjects were fed 7.5 g NSC/kg BW/day to maintain a fat body condition. Safety was assessed through daily monitoring, veterinary examination, and the measurement of fasting blood glucose, biochemistry and haematology. Efficacy at reducing post-prandial hyperinsulinemia was assessed using a diet challenge every 8 wk. during treatment and 4 wk. after withdrawal.

**Results:**

Velagliflozin was well accepted by all subjects and caused no adverse effects or hypoglycaemia. Post-prandial serum insulin (insulin C_max_) did not change significantly in the control animals over the entire study period (*P* = 0.101). In contrast, insulin C_max_ (mean ± SE) concentrations fell over time in the velagliflozin-treated group from 205 ± 25 μIU/mL in wk. 0, to 119 ± 19 μIU/mL (*P* = 0.015) and 117 ± 15 μIU/ml (*P* = 0.029) after 8 and 16 wk. of treatment, respectively. Although the insulin C_max_ in this group was not significantly lower than in controls at wk-8 (*P* = 0.061), it was lower at wk-16 (*P* = 0.003), and all 12 treated ponies were below the previously-determined risk threshold for laminitis at this time. After 4 wk. withdrawal, the insulin C_max_ returned to 199 ± 36 μIU/mL in the treated group, with no rebound effect.

**Conclusions:**

Velagliflozin appears to be a promising and safe treatment for equine insulin dysregulation, bringing post-prandial insulin concentrations below the laminitis risk threshold, albeit without normalising them.

**Electronic supplementary material:**

The online version of this article (10.1186/s12917-019-1811-2) contains supplementary material, which is available to authorized users.

## Background

Insulin dysregulation, including hyperinsulinemia and insulin resistance, is a core component of equine metabolic syndrome (EMS), which is associated with the development of the painful equine hoof condition laminitis [1]. High concentrations of insulin have been shown to induce laminitis experimentally, both after exogenous insulin administration [2, 3] and when driven by a high carbohydrate diet [4]. Hyperinsulinemia is a prospective and predictive laminitis risk factor [5, 6], and may be present in clinically laminitic, as well as previously laminitic ponies [7]. Insulin dysregulation can be exacerbated if insulin resistance occurs as a counter-regulatory response to chronic post-prandial hyperinsulinemia [8]. Hence, reducing hyperinsulinemia per se, could be the key to treating EMS and preventing laminitis.

At present, there are no registered pharmacological agents for reducing hyperinsulinemia in horses or ponies. Treatment options are confined to pasture restriction and/or the provision of a diet low in non-structural carbohydrates (NSC) [9, 10]; and exercise when physically possible to improve insulin sensitivity, though not effective in all animals [11–14]. Metformin and levothyroxine are currently recommended as adjuncts to management practices in treating insulin dysregulation, although they are un-registered and studies show conflicting results [15]. Novel therapies to reduce insulin secretion have been suggested, such as incretin receptor antagonists, but these are not yet available [16–18].

Sodium-glucose linked transport-2 (SGLT-2) inhibitors such as velagliflozin may provide a solution to address the medical need for an insulin reducing agent to treat insulin-dysregulation associated with EMS, and hence reduce the risk of insulin-associated laminitis. By reducing renal glucose reabsorption, SGLT-2 inhibitors promote urinary glucose excretion and have been shown to decrease excessive insulin secretion in response to hyperglycaemia in humans [19]. Recently, a proof of concept study demonstrated that velagliflozin reduces insulin concentrations and prevents laminitis in insulin-dysregulated ponies challenged with a high NSC diet for 18 d [20]. The treatment was given for a total of 39 d, and the results prompted the present study into the long-term efficacy and safety of velagliflozin; including an investigation of the compound’s effect on post-prandial hyperinsulinemia, insulin resistance, body weight, leptin concentrations and haematology/biochemistry parameters.

Hence, the first objective of this study was to investigate if a 16-week (wk) period of daily treatment (112 days) with velagliflozin would cause a sustained reduction in the hyperinsulinemic response to oral carbohydrates, or a change to insulin sensitivity, in insulin-dysregulated ponies The second objective was to identify any adverse effects (including changes in haematology and biochemistry) associated with treatment, or after treatment during a four-wk withdrawal period. The third objective was to determine if velagliflozin caused any changes in body weight or leptin concentrations.

## Results

### Selection and enrolment

The morphometric measurements of enrolled ponies did not differ between groups (Table 1). One control pony developed laminitis within the first month of the study and was removed from the trial. A second control pony was removed in the first month of the study due to intractable behavioural issues. As such, the data collected from these ponies are not included in any further results. Two additional control ponies who received the same pre-study procedures and testing were substituted for the purpose of monitoring changes in the response to oral carbohydrates but were not subjected to the combined glucose insulin tolerance test (CGIT).

**Table 1:** Pre-study signalment and morphometric measurements (mean ± SE, or median and range) of ponies enrolled in a study on the effects of velagliflozin

Measure	Control	Treated	*P* value
Age (yr)	16.03 ± 1.03	15.4 ± 1.4	0.709
Height (in)	42.3 ± 2.4	41.2 ± 2.4	0.748
Weight (kg)	227 ± 41	217 ± 22	0.291
Sex	8 F, 4 M	6 F, 6 M	0.408
BCS^a^	7 (6–9)	7 (6–9)	0.937
CNS^b^	3 (3–5)	3 (2–5)	0.808
n	12	12	

^a^*BCS*: Body Condition Score

^b^*CNS*: Cresty Neck Score

In terms of pituitary *pars intermedia* dysfunction (PPID), 2/12 control ponies and 1/12 treated ponies were diagnosed positive for the disease (*P* = 0.537). No pony underwent treatment for PPID with pergolide mesylate, or to our knowledge, had been treated prior to the experiment. The data for these PPID ponies are included in the results as PPID is a common condition in older ponies with EMS. Furthermore, removing these animals from the analysis did not alter the conclusions of the study. This group was not analysed as a separate cohort, as the number of ponies in this category was too low to draw reliable conclusions.

### Insulin and glucose responses to the oral high NSC diet challenge

**Fig. 1:**
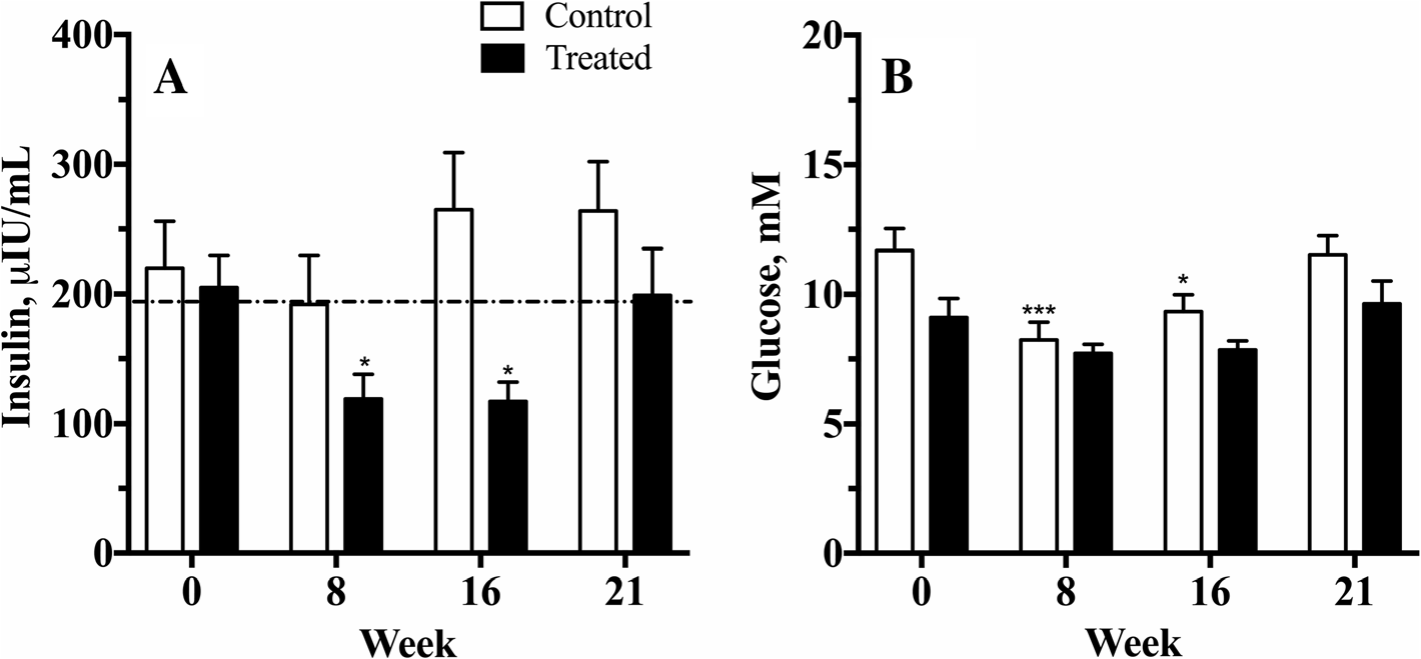
Maximum insulin and glucose responses (mean ± SE) measured in two groups of ponies after feeding a meal high in non-structural carbohydrates. Observations were made before the start of daily treatment with velagliflozin (0.3 mg/kg BW) or placebo (wk-0), during the middle and end of the treatment phase (wk 8 and 16), and 4 wk. after treatment ceased (wk-21). The maximum values represent either the 2 or 4 h measurement post-feeding. The dotted line represents a putative risk threshold for the development of insulin-induced laminitis. *P < 0.05 and ***P < 0.001 compared to corresponding group wk-0 pre-treatment values

The ponies treated with velagliflozin showed a decrease in their maximum insulin response to the diet challenge of 42% (*P* = 0.015) and 43% (*P* = 0.029) after 8 and 16 wk. of treatment, respectively, compared to the concentration observed in wk-0 (Table 2 and Fig. 1). This decrease had disappeared 4 wk. after therapy was withdrawn (wk-21), when insulin concentrations in the treated group had returned to within 3% of their pre-treatment values (*P* = 0.998). Importantly, no re-bound withdrawal response was observed. In comparison, the controls had no significant change in terms of either an increase or a decrease in their insulin C_max_ over the study period, (*P* = 0.101; Fig. 1). Comparing the maximum insulin response between control and treated groups over the entire study period revealed a significant effect of time (*P* = 0.006), no significant effect of treatment (*P* = 0.061) and a time x treatment interaction (*P* = 0.030). Further post-hoc testing revealed that after 16-wk of velagliflozin the treated group had significantly lower C_max_ insulin concentrations compared to the control group (*P* = 0.003).

**Table 2:** The frequency of ponies over the laminitis risk threshold of insulin C_max_ > 195 μIU/mL as measured after feeding a challenge diet high in non-structural carbohydrates to control and velagliflozin (treated) ponies

	Wk-0	Wk-8	Wk-16	Wk-21
Control (*n* = 12)	5/12 (42%)	6/12 (50%)	8/12 (67%)	9/12 (75%)
Treated (*n* = 12)	6/12 (50%)	2/12 (17%)	0/12 (0%)**	6/12 (50%)

***P < 0.001 compared between-groups at each week using Fisher’s exact test

The frequency of ponies with C_max_ insulin concentrations above the laminitis risk threshold of > 195 μIU/mL as previously determined for this diet [4] is presented in Table 2. Both groups were similar at wk-0, with no decline in the control group as the study progressed. In comparison, the number of at-risk ponies in the velagliflozin-treated group decreased over the study period, and after 16 wk. of velagliflozin treatment no pony was above this risk threshold. After the withdrawal of velagliflozin, the number of ponies at-risk returned to the pre-treatment level.

The glucose C_max_ was unchanged over the 21-wk treatment period in the velagliflozin group (*P* = 0.132) as shown in Fig. 1. Unexpectedly, the control group showed a decrease in glucose C_max_ at wk-8 (*P* = 0.0008) and at wk-16 (*P* = 0.043), but this had increased again at the end of the study, and was unchanged at wk-21 compared to the wk-0 values (*P* = 0.139). Comparing the maximum glucose response between control and treated groups over the entire study period revealed a significant effect of time (*P* < 0.0001), no time x treatment interaction (*P* = 0.227) and no significant treatment effect (*P* = 0.075). Post-hoc testing failed to identify any significant differences between the two groups over the four testing wk. (*P* > 0.121 all cases).

### Insulin sensitivity

The insulin concentrations measured during the CGIT were not different between the control and treated groups in wk-0, and nor did this change over the study period (*P* = 0.685), or within each group over time (*P* = 0.344), as shown in Table 3. Insulin resistance (IR) was suspected if the 45-min insulin concentration was > 100 μIU/mL [21]. This was diagnosed in a minority of animals in both the control and treated groups, with no difference between the groups in the frequency of IR at any time (*P* > 0.277 all cases).

**Table 3:** Insulin concentrations (geometric mean, 95% CI) measured during a combined-insulin-glucose tolerance test in control and velagliflozin (treated) ponies

	Wk-0	Wk-16	Wk-21
Insulin 0 min, μIU/mL			
Control, *n* = 10	5.4 (3.1 – 9.4)	5.8 (3.9 – 8.6)	8.7 (5.8 – 13.1)
Treated, *n* = 12	6.4 (2.7 – 15.3)	4.9 (3.4 – 7.1)	6.7 (4.6 – 9.6)
Insulin 45 min, μIU/mL			
Control, *n* = 10	73.5 (40.7 – 132.8)	61.5 (41.5 – 91.2)	73.2 (46.5 – 115.4)
Treated, *n* = 12	95.4 (52.9 – 172.1)	96.2 (54.0 – 171.4)	83.0 (55.6 – 123.8)
Insulin 75 min, μIU/mL			
Control, *n* = 10	27.7 (16.7 – 46.1)	21.1 (12.3 – 36.0)	20.9 (10.5 – 41.5)
Treated, *n* = 12	33.1 (18.3 – 59.8)	28.9 (16.2 – 51.5)	26.4 (15.0 – 46.5)
Number insulin resistant†			
Control, *n* = 10	4/10 (40%)	3/10 (30%)	2/10 (20%)
Treated, *n* = 12	5/12 (42%)	5/12 (42%)	5/12 (42%)

**†**Insulin resistance classified if insulin concentration > 100 μIU/mL at 45 min time point

**Fig. 2:**
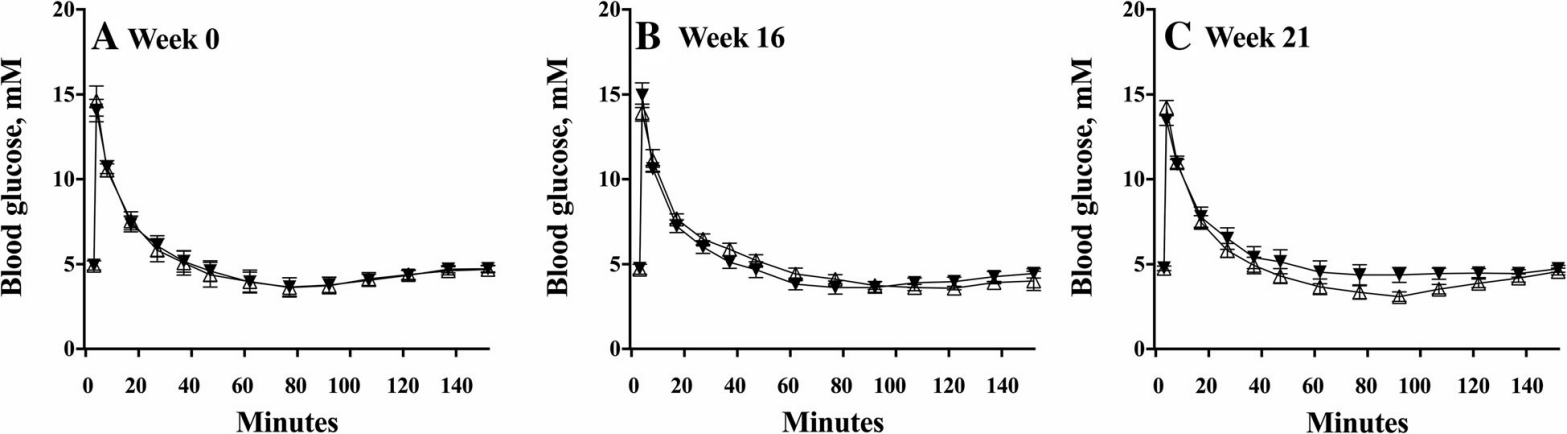
Glucose concentrations (mean ± SE) during a series of three combined glucose-insulin tolerance tests. Tests were conducted over 150 min, one wk. before treatment (A), after 16 wk. of daily treatment (B) and after 4 wk. of treatment withdrawal (C) in ponies given placebo (control; closed triangles) or velagliflozin (treated; open triangles) daily

The data demonstrate that the glucose responses mirrored those of the insulin responses, with no difference in glucose concentrations during the CGIT between groups, either before treatment (wk-0), during treatment (wk-16), or after treatment was withdrawn (wk-21; Fig. 2).

### Bodyweight and condition scores

Overall, there was no significant change in BW over the study period in either the control (*P* = 0.250) or treated groups (*P* = 0.158). Similarly, body condition and cresty neck scores showed no significant changes over the study, either within or between groups (*P* > 0. 281 all cases; Table 4).

**Table 4:** Bodyweight (geometric mean, 95% CI), body condition score and cresty neck score (median, range) in control and velagliflozin (treated) ponies^a^

	Wk-0	Wk-16	Wk-21
Bodyweight, kg			
Control	201 (141–287)	206 (145–293)	206 (144–294)
Treated	206 (166–256)	209 (169–259)	212 (170–263)
Body condition score			
Control	7 (6–9)	7 (6–8.5)	7.5 (5.5–8.5)
Treated	7 (6–9)	7.5 (6–8.5)	8 (5.5–9)
Cresty neck score			
Control	3 (3–5)	3.5 (2.5–5)	3.5 (2.5–5)
Treated	3 (2–5)	3.75 (2.5–4.5)	4.5 (2–4.5)

^a^Measurements were observed before treatment (wk-0), 16 wk. after treatment, and 4 wk. after withdrawal in ponies given placebo (*n* = 10) or velagliflozin (*n* = 12)

### Safety: Assessing the risk of hypoglycaemia

During the study, basal blood glucose was measured in all ponies after a 13 h overnight fast in both wk-8 and wk-16 of treatment. Based on the normal reference range for equine blood glucose of 3.4–7.4 mM [25], no hypoglycemia occurred, and the lowest glucose concentration measured in any pony was 3.9 mM. The control basal glucose values (median, range) were 4.4 (4.1–5.7) mM at wk-8 and 4.4 (4.0–5.1) mM at wk-16, and the values for the treated group were similar at 4.3 (3.9–5.5) mM in wk-8 (*P* = 0.230), and 4.3 (3.8–8.3) mM in wk-16 (*P* = 0.280).

### Clinical examinations and daily health monitoring

No adverse health effects were detected during the study attributable to an adverse reaction or side-effect of velagliflozin. Polydipsia and polyuria was not noted during the study. In addition, ponies were monitored twice-daily for laminitis by trained observers after the withdrawal of velagliflozin in case of rebound hyperinsulinemia, but clinical laminitis did not occur.

### Biochemistry, haematology and leptin

Overall, biochemistry and haematology results were unremarkable in both groups, with the full data presented in Additional file 1. A within-group analysis was conducted over the 21-wk period, and any changes are identified.

The geometric mean (95% CI) concentrations for leptin in the treated group (*n* = 12) were 20.1 (14.4–23.5), 18.7 (14.9–23.5) and 21.5 (14.1–33) ng/mL at wk-0, 16 and 21, respectively. Corresponding values for the control group (*n* = 10) were 20.3 (13.9–29.8), 21.9 (15.4–31.8) and 23.5 (15.2–36.4) ng/mL, respectively. There was no significant effect of time (*P* = 0.552), treatment (*P* = 0.574) or interaction (*P* = 0.852) on leptin concentrations over the 21-wk of the study.

## Discussion

The study investigated the efficacy and safety of velagliflozin over 16 wk. of treatment, and a four wk. withdrawal period in 12 insulin-dysregulated ponies. No adverse effects were identified, and the therapy was well accepted, as well as efficacious in reducing the hyperinsulinemic response to oral NSC.

The application of sodium-glucose linked transport inhibitors within the veterinary field is new. It is encouraging that no clinical signs of adverse effects were seen during daily monitoring by the researchers, or during regular physical examinations conducted by experienced veterinarians. The absence of any remarkable changes in blood biochemistry and haematology, attests further to the safety of this compound.

In terms of SGLT-2 safety, their mode of action in promoting glucosuria raises a concern about the inherent risk of hypoglycaemia. Importantly, no hypoglycaemia was evident during the present study, either clinically, or during the measurement of basal blood glucose after a 13 h overnight fast. This is consistent with our previous results [20], and with the use of SGLT-2 inhibitors in humans, where the risk of hypoglycaemia is considered low, as the renal threshold for glucose excretion is not lowered to the point of the hypoglycemic threshold [26]. Additionally, in humans, hepatic gluconeogenesis protects against hypoglycaemia, and it was recently concluded in a velagliflozin study in felines that endogenous glucose production likely balanced the action of velagliflozin to maintain euglycemia [26, 27].

A transient decrease in C_max_ blood glucose occurred in the control group at wk-8 and wk-16, but not in the velagliflozin-treated group. No confounding factors could be identified to explain this difference, although numerically the control group had slightly higher glucose concentrations in wk-0 than the treated animals (Fig. 1). It is possible that endogenous glucose production in velagliflozin-treated animals prevented a reduction in C_max_ glucose, and this could explain the lack of association between insulin and glucose changes in this group (Fig. 1). This mechanism should be investigated further, including the quantification of glucagon concentrations in velagliflozin-treated equids.

In terms of efficacy, the reduction in hyper-responsiveness to oral carbohydrates was convincing. In particular, our previous study identified a clear relationship between the speed of onset and the frequency of laminitis in ponies that were fed the same diet challenge as used in the present study, for up 18 d. A risk threshold for laminitis was identified, when the insulin C_max_ exceeded 195 μIU/mL in response to the diet challenge meal (Fig. 1) [4]. Importantly, velagliflozin reduced the insulin response to well below this threshold in all treated ponies after 16 wk. of treatment (Table 2). Although post-prandial insulin concentrations were not normalized, these findings are strongly encouraging. Additionally, the diet challenge meal provided considerably more NSC (3.8 g NSC/kg BW) than the oral sugar test (OST) that is recommended by the Equine Endocrinology group for the diagnosis of insulin dysregulation [15]. The diet challenge was used to assess insulin dysregulation in this study as access to corn syrup in Australia is very limited. Additionally, the diet challenge has been shown to provide clear demarcation between ponies who did and did not develop laminitis when fed the diet for up to 18 d, which was useful when considering velagliflozin as a potential laminitis preventive agent [4]. Nevertheless, the diet challenge is not recommended for routine clinical use due to a greater risk that laminitis may be incited by this test.

The present study has also established that the effects of velagliflozin are not long-lasting, as the insulin concentrations returned to within 3% of the pre-trial measurements after a 4-wk withdrawal. The absence of any rebound increase in the insulin response to the diet challenge is a promising finding, although we can-not rule out the possibility of a short-term rebound effect within the 4-wk period between diet challenge tests.

It is noteworthy that velagliflozin did not affect insulin resistance per se in this study, with no change in CGIT measurements of glucose or insulin concentrations over time, or in the number of ponies classified as IR according to the test. Previously, we have speculated that IR in horses is driven by chronic hyperinsulinemia, which results from excessive glucose absorption from the gut [8]. However, in the present study, the hyperinsulinemic response to oral NSC was reduced in the treated ponies, but IR remained unchanged. Thus, the present results suggest that more research is needed to better understand the relationship between hyperinsulinemia and IR, with the possibility that different modes of treatment may be necessary to suit different forms of insulin dysregulation. Additionally, we acknowledge that the interpretation of the CGIT results presented in this paper is complicated by the use of a chemiluminescent insulin assay, whereas radioimmunoassays have been used in the reference studies cited [21, 28]. It has been shown when comparing these types of assays that results are not consistent [29, 30]. Additionally, glucose values were not used to generate a binary classification of IR (positive or negative), due to recently published data concerning poor specificity as a diagnostic test [21]. Hence, the CGIT data presented here should be interpreted with caution.

A factor important to the interpretation of the present results, is that the ponies were maintained on a high NSC diet throughout the study, in order keep the animals in a ‘fat’ body condition. This diet provided considerably more energy and NSC than is currently recommended for feeding insulin-dysregulated horses [31–33], and indeed, laminitis was incited in one control. This diet may have also mitigated any effect of velagliflozin on BW, which was unchanged for the study duration. The results in this study contrast with those from some human and rodent experiments, where SGLT-2 inhibitors have caused a modest weight loss [34–36]. Despite the limitations of this diet, it did provide a useful insight into the likely effects of velagliflozin as a treatment for inactive (stabled), over-fed, insulin-dysregulated ponies, and the fact that the compound was efficacious in this situation is promising for future clinical trials.

Leptin concentrations were moderate to high in both groups of ponies according to the current test interpretations, although as a technical caveat, comparisons to other published data may be difficult as the antibodies of the test were changed in 2016 [37]. The data seem to be consistent with the fat body condition of the ponies in the present study, and as leptin has been shown to be proportional to fat mass and BW it may be unsurprising that leptin concentrations were unchanged throughout this study, as BW did not change [38, 39]. However, other studies in horses have shown that feed restriction was associated with a decrease in leptin concentrations [40], and conversely, our ponies were fed 1/3 of the diet challenge meal in the two days prior to diet challenge testing to allow taste adjustment, hence this extra meal may have affected leptin concentrations. Studies in other species have shown that SGLT-2 inhibitors decrease leptin concentrations [41, 42], and the decrease may be independent of significant weight loss [43]. In another study performed by the authors, leptin concentrations decreased after 16 wk. of velagliflozin treatment irrespective of bodyweight (unpublished data). Variability between ponies in terms of breed, height and weight, and also the relatively small sample size of the two groups in this study could additionally explain the lack of difference seen in the present study. Within human literature, there is growing evidence that leptin is not merely a marker of obesity, but an important pro-inflammatory adipokine involved in the pathophysiology of metabolically induced cardiovascular and renal disease [44]. Hence, the effect of velagliflozin on the relationship between bodyweight, biologically active fat deposits and leptin concentrations should be investigated further.

Besides the relatively small sample size of the two groups, further limitations of the study include the use of a CGIT to assess insulin sensitivity, which might have been better evaluated using a euglycemic hyperinsulinemic clamp, or a frequently-sampled intravenous glucose tolerance test [45]. Furthermore, if it had been logistically possible, it would have been useful to conduct urinalysis, and also to quantify the water intake and urinary output in this study.

Lastly, as the ponies were stabled and fed a set high NSC diet, the relevance of these results to animals at pasture could be questioned. Conversely, it could be argued that NSC intake is most difficult to limit in a grazing situation, and coupled with a genetic predisposition towards insulin dysregulation, this is the fundamental reason why so many animals develop laminitis. As endocrinopathic laminitis is the most common form of the disease [46], future studies should examine velagliflozin under grazing conditions.

## Conclusions

This study has built on earlier work in demonstrating the efficacy and safety of velagliflozin in insulin-dysregulated ponies over 16 wk. of treatment and over 5 wk. after drug withdrawal. Velagliflozin reduced post-prandial hyperinsulinemia, and although this was not completely normalized in response to the diet challenge test, 16 wk. of treatment all the ponies that received velagliflozin were below the laminitis-risk threshold for this diet. The present results confirm that velagliflozin warrants further investigation as a much needed treatment for EMS and a preventive for insulin-associated laminitis in horses and ponies.

## Methods

### Animal selection

The ponies used in this study were part of a large herd owned by Queensland University of Technology. Twenty-four unrelated mares and geldings were selected from the herd over a 6-wk period based on their degree of insulin dysregulation, which was assessed according to their insulin response to a high NSC diet challenge. The diet challenge test was developed in a previous study [4] and entailed an overnight fast, followed by a morning (08:00 h (h)) meal of mixed sweet feed containing: 0.33 g/kg bodyweight (BW) dextrose, 2.75 g/kg BW molasses, 2.64 g/kg BW roasted micronized oat flakes^1^ and 1.65 g/kg BW lucerne (alfalfa) chaff; delivering a total of 3.8 g NSC/kg BW. The ponies became accustomed to eating the diet challenge after 1/3 of the meal was presented each day for two days prior to the actual test. On the test day, a basal sample was collected at 0 h, the ponies were then fed immediately, and two further samples were collected 2 h and 4 h after feeding, to measure blood glucose and serum insulin concentrations as described below. The ponies ate with variable speed, but all finished their meals within the 4-h period.

Ponies were enrolled if their highest insulin concentration (C_max_) during a screening diet challenge was > 90 μIU/mL either 2 h or 4 h after feeding. The ponies were paired based on their insulin C_max_, and then allocated at random by coin toss into either control (*n* = 12) or treatment groups (n = 12). The signalment and morphometric data for each group are shown in Table 1. Additionally, enrolled breeds included Shetland pony, Miniature horse, Miniature pony, Welsh mountain pony, Connemara, Palouse pony, Arabian pony and Australian pony, plus mixed derivatives of these breeds. One Quarter horse was enrolled, in the control group, which fitted the EMS classification of having generalised and regional adiposity, as well as insulin dysregulation and a predisposition to laminitis.

### Animal management

Pre-experiment health management procedures included Hendra virus vaccination, hoof trimming, pregnancy testing, veterinary dental treatment and oral parasiticide administration. During the selection phase, the ponies were kept on a 30-acre grass paddock and received supplementary feed comprising 1% BW lucerne hay and 0.5% BW complete extruded cubes.^2^

One wk. prior to starting, and for the duration of the experiment, the ponies were kept in individual stables during the day (07:00 h – 17:00 h), and a communal 2 acre dry-lot at night. During the experiment, the diet was 1.5% BW lucerne/grass hay blend, 0.5% BW lucerne chaff, 0.5% BW extruded cubes^2^, plus a balanced vitamin and mineral supplement^3^ dosed according to manufacturer’s guidelines for inactive ponies. The diet was split into two daily rations fed at 08:00 h and 15:00 h, with no feed overnight. At all times throughout the study ponies had access to ad libitum water. The diet delivered a total daily allowance of 2.5% feed/kg BW and 7.5 g NSC/kg BW. It was designed to be highly calorific and to maintain fatness, consistent with the ponies’ pre-existing body condition (Table 1). Diet analyses were performed by Feed Quality Service^4^ and the results are shown in Table 5.

**Table 5:** Analysis of a diet fed to ponies during a 21-wk study of the effects of velagliflozin

Average feed value	lucerne chaff	extruded cubes	lucerne/grass blend hay
Dry Matter, %	87.4	92.5	86.9
Neutral Detergent Fiber, %	39	11	50
Acid Detergent Fiber, %	30	3	32
Crude Protein, %	26.1	15	18.2
Dry Matter Digestibility, %	71	85	65
Digestible Organic Matter in the Dry Matter, %	67	84	62
Inorganic Ash, %	10	8	9
Organic Matter, %	90	92	91
Metabolizable Energy, MJ/kg	10.6	12.9	9.5
Crude Fat, %	1.8	1.7	2.1
Total Non-Structural Carbohydrates, %	23	64.3	21

### Treatments

For logistical reasons the experiment was conducted using two replicates, staggered by 2 wk., with six control, and six treated ponies starting simultaneously. Velagliflozin was mixed in a carrier solution (60 μL/kg BW) and administered daily at 08:00 h, at a dose of 0.3 mg/kg BW by mouth for 16 wk., then withdrawn, with clinical observations continuing for 5 wk. until the study ended in wk-21. Control ponies received carrier solution alone (60 μL/kg BW) for 16 wk. The dose of velagliflozin was based previous in-house studies at Boehringer Ingelheim [20].

### Blood sampling and analysis

During the 21-wk experiment, each group received a diet challenge as described above, to assess their insulin status at the following stages: screening, before treatment (wk-0), during treatment (wk-8), at the end of the treatment period (wk-16), and after 4 wk. of drug withdrawal (wk-21). Samples for blood glucose and serum insulin were collected by jugular venipuncture at 0 h (before meal) and 2 h and 4 h post-feeding. In this paper, the C_max_ is used as a relative term only, representing the higher of the two post-prandial measurements.

Samples for insulin analysis (4 mL) were collected into serum vacutainers and assayed at a commercial laboratory^5^ using a chemiluminescent assay with the ADVIA Centaur and ADVIA Centaur XP systems. The validation data supplied by the manufacturer for human samples for this assay have been previously published [4]. Glucose concentrations were measured immediately in drops of whole blood using a portable glucometer^6^ previously validated against laboratory samples [4].

An additional 4 mL of blood was collected into EDTA vacutainers at these sampling times, which was chilled on ice prior to centrifugation. The plasma was harvested and stored at -20 °C for subsequent biomarker analysis. Basal (0 h) concentrations of leptin were measured in plasma samples collected in wk-0, 16 and 21. The Millipore Multi-Species Leptin Radioimmunoassay^7^ was used to quantify leptin concentrations according to manufacturer’s directions. Our in house intra- and inter-assay coefficients of variation (CV%) were 15.1, and 9.7%, respectively. Samples of EDTA-plasma for haematology and serum for biochemistry analysis were taken during wk. 0, 16 and 21, and were analysed on the same day by a commercial laboratory^5^. These data are shown in Additional file 1.

In addition to the diet challenge a CGIT was performed to assess insulin sensitivity. This was done prior to treatment (wk-0), in wk-16 of the study, and 4 wk. after velagliflozin withdrawal (wk-21), two to four days after the diet challenge test. Intravenous jugular catheters were fitted the night before the CGIT was performed, and ponies were fasted overnight. On the day of the test, glucose^8^ was given as an intravenous bolus at a dose of 150 mg/kg BW, followed immediately by insulin^9^ at 0.1 IU/kg BW. Blood samples (0.1 mL) were collected at 0, 1, 5, 15, 25, 35, 45, 60, 75, 90, 105, 120, 135 and 150 min for immediate blood glucose analysis. Samples for insulin analysis were collected into serum vacutainers at 0, 45 and 75 min, and were analysed by a commercial laboratory as described above. Insulin resistance (IR) was suspected if the 45-min insulin concentration was > 100 μIU/mL [21].

Pituitary *pars intermedia* dysfunction (PPID) was diagnosed using a combination of clinical signs indicative of the disease (hirsuitism, muscle wastage, abnormal fat distribution and polyuria/polydipsia) and basal adrenocorticotrophic hormone (ACTH) concentration. Blood was collected into EDTA vacutainers at 08:00 h after an overnight fast. After cooling on ice for 10 min, the samples were centrifuged and plasma transferred into Eppendorf tubes and frozen at − 4 °C before shipping for analysis at a commercial laboratory using an Immulite 2000 chemiluminescence assay.^10^ Basal ACTH concentrations were measured in May in all ponies (autumn in the southern hemisphere) and were considered elevated if they exceeded the seasonally-adjusted cut-off of > 80 pg/mL [22].

### Clinical measurements

Clinical examinations of the subjects were conducted in wk. 0, 1, 8, 16, 19 and 21. Assessment included BW, girth circumference, body condition score (BCS) [23] and cresty neck score (CNS) [24] allocated by two un-blinded trained assessors and averaged. Veterinary clinical examination was conducted by blinded consultant veterinarians and included assessment of demeanour, thoracic and abdominal auscultation, heart rate, respiratory rate, temperature, forelimb digital pulse palpation, capillary refill time, mucous membrane colour, skin turgor, lymph node palpation, lameness examination and visual inspection for any abnormalities.

### Data analysis

The data were subjected to a Shapiro-Wilk test for normality, and if necessary, they were log transformed and re-tested. Data fitting a normal distribution were analysed by one-way Analysis of Variance (ANOVA) or two-way ANOVA (with or without repeated measures), using Dunnet’s, Tukey’s, or Holm-Sidak’s post-hoc testing to separate the means; or paired/unpaired t-tests as appropriate. Likewise, non-parametric data were analysed using the Mann-Whitney, Kruskal-Wallis or Friedman tests, with Dunn’s multiple comparison test for post-hoc analysis. A Chi-squared test was used to determine differences in frequencies between groups of ponies, for example the presence or absence of PPID and male vs female enrolment. A Fisher’s exact test was used to determine differences between control and treated groups in the number of animals above the laminitis risk threshold of C_max_ insulin > 195 μIU/mL, as measured during the diet challenge and previously determined for this test [4]. Data are presented as mean ± SE, geometric mean and 95% confidence interval, or median (range) as appropriate, depending on normality. All analyses were made using Prism version 7^11^ and Sigmaplot version 13^12^ statistical software programs. Significance was set at *P* < 0.05.

### Fate of the animals

All the ponies were re-homed at the end of the study, adopted by experienced horse owners who are members of the local/regional community.

## Additional file

Additional file 1: Haematology and biochemistry results. Haematology and biochemistry results (median, range) measured pre-study (week-0), after four months of treatment (week-16), and 5 weeks after treatment withdrawal (week-21) in control (*n* = 10) and velagliflozin-treated ponies (*n* = 12). Data were analysed within-groups using a repeated measures one-way ANOVA or the non-parametric equivalent Friedman test. (DOCX 32 kb)

## Abbreviations

ACTH: Adrenocorticotrophic hormone
ANOVA: Analysis of variance
BCS: Body condition score
BW: Body weight
CGIT: Combined glucose insulin tolerance test
CNS: Cresty neck score
CV: Coefficient of variation
EDTA: Ethylenediaminetetraacetic acid
EMS: Equine metabolic syndrome
h: Hours
insulin C_max_: The higher of two post-prandial serum insulin concentrations
IR: Insulin resistance
IU: International units
NSC: Non-structural carbohydrates
PPID: Pituitary *pars intermedia* dysfunction
QUT: Queensland University of Technology
SGLT-2: Sodium-glucose linked transport-2
wk.: Week

## Footnotes

1 Riverina, Milton, Queensland, Australia

2 Prydes Easiresponse, Gunnedah, New South Wales, Australia

3 Equilibrium, Equiaustralia, Loganholme, Queensland. Australia

4 New South Wales Department of Primary Industries, Wagga Wagga, New South Wales Australia

5 QML Pathology, Murrarie, Queensland, Australia

6 Accu-chek, Roche Diagnostics, Castle Hill, New South Wales, Australia

7 EMD Millipore Corporation, Billerica, MA, USA

8 Baxter Healthcare, Old Toongabbie, New South Wales, Australia

9 Humulin-R, Eli Lilly, Melrose Park, New South Wales, Australia

10 Vetpath Laboratory Services, Ascot, Western Australia, Australia

11 GraphPad Software, La Jolla, California, USA

12 Systat Software Inc., San Jose, California, USA
