# Supplementary material for: The efficacy and safety of velagliflozin over 16 weeks as a treatment for insulin dysregulation in ponies
Source: BMC Vet Res. 2019 Feb 26;15:65. doi: 10.1186/s12917-019-1811-2 (PMC6390376; doi:10.1186/s12917-019-1811-2)
Supplement: Supplementary file 1 — Haematology and biochemistry results. Haematology and biochemistry results (median, range) measured pre-study (week-0), after four months of treatment (week-16), and 5 weeks after treatment withdrawal (week-21) in control (n = 10) and velagliflozin-treated ponies (n = 12). Data were analysed within-groups using a repeated measures one-way ANOVA or the non-parametric equivalent Friedman test. (DOCX 32 kb) [file 12917_2019_1811_MOESM1_ESM.docx]

**Additional file 1.** Haematology and biochemistry results (median, range) measured pre-study (week-0), after four months of treatment (week-16), and 5 weeks after treatment withdrawal (week-21) in control (n = 10) and velagliflozin-treated ponies (n = 12). Data were analysed within-groups using a repeated measures one-way ANOVA or the non-parametric equivalent Friedman test.

|  | **Control group** | | |  | **Treated group** |  |
| --- | --- | --- | --- | --- | --- | --- |
|  | **Week 0** | **Week 16** | **Week 21** | **Week 0** | **Week 16** | **Week 21** |
| **HCT (0.31 - 0.49)** | 0.35 (0.28 – 0.46) | 0.38 (0.3 – 0.51) | 0.35 (0.27 – 0.44) | 0.33 (0.24 - 0.4) | 0.36 (0.3 – 0.42) | 0.34 (0.27 – 0.44) |
| **Hb (95 - 155) g/L** | 112 (89 – 143) | 117 (96 – 158) | 111 (88 – 139) | 109 (78 – 130) | 111 (94 – 128) | 107 (88 – 143) |
| **RCC (6.0 - 10.5) 10^12^/L** | 6.5 (5.2 – 8.6) | 6.7 (5.5 – 9.5) | 6.5 (4.9 – 8.2) | 6.1 (4.5 – 7.4) | 6.4 (5.3 – 7.6) | 6.2 (4.7 – 8.5) |
| **MCV (36 - 59) fL** | 53 (46 – 57) | 54 (51 – 60) | 54 (49 – 60) | 55 (49 – 56)^a^ | 56 (52 – 60) | 56 (53 – 61)^b^ |
| **MCH (13 - 19) pg** | 17 (16 – 19) | 17.0 (16 – 19) | 17 (16 – 19) | 18 (16 – 19) | 17.5 (17 – 19) | 18 (17 – 19) |
| **MCHC (340 - 380) g/L** | 321 (312 – 355) | 314 (309 – 326) | 319 (306 – 355) | 326 (311 – 359)^a^ | 315 (307 – 325)^b^ | 317 (307 – 325)^a,b^ |
| **WBC (5.8 - 11.0) x10^9^ /L** | 8.6 (6 – 14.4) | 8.2 (5 – 9.9) | 7.8 (4.4 – 10.2) | 9.1 (5.1 – 11.2) | 8.4 (4.4 – 13.3) | 8.2 (4.8 – 12.9) |
| **Neutrophils (2.5 - 7.0) x10^9^/L** | 5 (2.8 – 7.6) | 3.8 (1.8 – 5.6) | 4.6 (1.8 – 6.4) | 4.4 (2 – 6.3) | 4 (2.1 – 11) | 3.6 (2.5 – 8.9) |
| **Lymphocytes (1.6 - 5.4) x10^9^/L** | 2.9 (1.8 – 6.2) | 3.4 (2.2 – 3.9) | 3.1 (1.8 – 4.2) | 3.7 (1.3 – 5.2) | 3.7 (1 – 5.9) | 3.5 (1.9 – 7.9) |
| **Monocytes (0.1 - 0.7) x10^9^/L** | 0.3 (0.1 – 0.5) | 0.2 (0 – 0.4) | 0.3 (0.1 – 0.5) | 0.2 (0.1 – 0.5) | 0.3 (0.1 – 0.4) | 0.2 (0 – 0.7) |
| **Eosinophils (0.10 - 0.50) x10^9^/L** | 0.4 (0.1 – 1.3) | 0.3 (0 – 1.6) | 0.3 (0 – 0.5) | 0.4 (0.1 – 0.9) | 0.3 (0 – 1.1) | 0.3 (0 – 1.5) |
| **Platelets (80 - 300) x10^9^ /L** | 209 (126 – 249) | 172 (132 – 217) | 172 (121 – 230) | 190 (141 – 261) | 186 (114 – 248) | 200 (146 – 304) |
| **Fibrinogen (2 - 4) g/L** | 3.7 (2.4 – 4.6) | 3 (2.3 – 4.3) | 3.6 (2.1 – 4.7) | 3.9 (2.3 – 4.7) | 2.4 (2 – 4.7) | 2.8 (2.3 – 4.6) |
| **Na (132 - 146) mmol/L** | 137 (133 – 138) | 137 (134 – 138) | 136 (134 – 138) | 136 (132 – 140)^a^ | 137 (132 – 140)^a,b^ | 137 (136 – 141)^b^ |
| **K (3.1 - 4.9) mmol/L** | 4 (3.6 – 5) | 4.5 (3.6 – 5.7) | 4.1 (3.6 – 8.5) | 4.1 (3.5 – 4.4) | 4.1 (3.2 – 5.3) | 4.0 (3.5 – 4.3) |
| **Cl (94 - 105) mmol/L** | 99 (95 – 102) | 100 (96 – 101) | 98 (95 – 101) | 99 (95 – 102)^a,b^ | 97 (94 – 102)^a^ | 100 (94 – 105)^b^ |
| **HCO3 (20 - 32) mmol/L** | 25 (24 – 26)^a^ | 25 (22 – 27)^a.b^ | 22 (19 – 26)^b^ | 26 (23 – 28)^a,b^ | 23.5 (18 – 26)^a^ | 24 (20 – 26)^b^ |
| **An Gap (4 - 17) mmol/L** | 17 (13 – 20) | 18 (14 – 20) | 20 (17 – 23) | 15 (13 – 20)^a^ | 21 (16 – 24)^b^ | 18 (15 – 24)^a,b^ |
| **Urea (4.4 - 7.8) mmol/L** | 5.2 (4.4 – 7.4) | 5.5 (4.5 – 7.2) | 5.3 (3.1 – 6.3) | 5.4 (4.2 – 8)^a^ | 5.9 (5 – 8.2)^a,b^ | 6.2 (4.3 – 8.7)^b^ |
| **Creatinine (55 - 135) umol/L** | 92 (74 – 135) | 91 (67 – 118) | 79 (71 – 115) | 81 (59 – 115) | 83 (56 – 146) | 90 (66 – 115) |
| **Bilirubin (12 - 62) umol/L** | 13 (8 – 32) | 13 (7 – 27) | 13 (9 – 25) | 9 (5 – 17) | 13 (5 – 34) | 10 (6 – 18) |
| **AST (1 - 450) U/L** | 303 (250 – 637) | 397 (279 – 546) | 341 (264 – 499) | 426 (299 – 618)^a,b^ | 449 (231 – 997)^a^ | 345 (223 – 455)^b^ |
| **GGT (1 - 49) U/L** | 18 (11 – 33) | 15 (12 – 34) | 16 (13 – 30) | 24 (15 – 50) | 21 (10 – 60) | 19 (11 – 26) |
| **ALP (1 - 280) U/L** | 187 (124 – 363)^a^ | 136 (89 – 209)^b^ | 143 (87 – 208)^b^ | 161 (122 – 288)^a^ | 129 (76 – 271)^b^ | 155 (95 – 304)^a^ |
| **Protein (58 - 72) g/L** | 70 (61 – 79) | 68 (65 – 74) | 72 (65 – 77) | 73 (68 – 78) | 70 (62 – 80) | 71 (63 – 80) |
| **Albumin (28 - 37) g/L** | 31 (26 – 34)^a,b^ | 32 (27 – 37)^a^ | 31 (24 – 35)^b^ | 29 (23 – 31)^a^ | 31 (26 – 36)^b^ | 29 (26 – 34)^a,b^ |
| **Globulins (25 - 42) g/L** | 40 (30 – 49) | 36 (30 – 46) | 39 (30 – 50) | 35 (37 – 52) | 40 (26 – 51) | 42 (29 – 51) |
| **Ca (2.50 - 3.30) mmol/L** | 3 (2.7 – 3.3) | 3.1 (2.8 – 3.2) | 3.2 (2.9 – 3.3) | 3.0 (2.9 – 3.2) | 3.0 (2.9 – 3.3) | 3.0 (2.8 – 3.3) |
| **PO4 (0.5 – 1.8) mmol/L** | 1 (0.6 – 1.2) | 0.9 (0.7 – 1.1) | 1 (0.7 – 1.4) | 1.1 (0.7 – 1.3) | 1 (0.6 – 1.1) | 1.1 (0.9 – 1.8) |
| **Creatine Kinase (1 - 648) U/L** | 389 (270 – 1073) | 373 (286 – 449) | 444 (293 – 609) | 393 (298 – 913) | 406 (197 – 838) | 437 (334 – 764) |
| **Mg (0.7 - 1.1) mmol/L** | 0.8 (0.6 – 1) | 0.8 (0.6 – 0.8) | 0.7 (0.4 – 1.0) | 0.8 (0.7 – 1) | 0.8 (0.6 – 0.9) | 0.8 (0.6 – 0.9) |
| **Cholesterol (1.9 - 2.9) mmol/L** | 2 (1.6 – 2.7) | 1.9 (1.4 – 2.1) | 1.8 (1.3 – 2.1) | 1.9 (1.2 – 2.1)^a^ | 2 (1.6 – 3)^b^ | 1.8 (1.4 – 2.8)^a,b^ |
| **Triglyceride (0.1 - 0.5) mmol/L** | 0.3 (0.1 – 0.6) | 0.5 (0.2 – 0.5) | 0.4 (0.2 – 0.5) | 0.2 (0.1 – 0.4)^a^ | 0.5 (0.3 – 1.4)^b^ | 0.4 (0.2 – 0.6)^a,b^ |

^a,b^ Medians in a row with different superscripts, P < 0.05.
